# Fostering ICT Competencies in Blended Learning: Role of Curriculum Content, Material, and Teaching Strategies

**DOI:** 10.3389/fpsyg.2022.758016

**Published:** 2022-07-04

**Authors:** Muhammad Azeem Ashraf, Javed Iqbal, Muhammad Irfan Arif, Muhammad Zaheer Asghar

**Affiliations:** ^1^Research Institute of Education Science, Hunan University, Changsha, China; ^2^Department of Education, University of Management and Technology, Lahore, Pakistan; ^3^Division of Education, University of Education, Lahore, Pakistan; ^4^Department of Teacher Education, University of Helsinki, Helsinki, Finland

**Keywords:** ICT-integrated curriculum contents, ICT-integrated curriculum materials, ICT-integrated teaching strategies, ICT competencies, blended learning

## Abstract

The study examined the direct and indirect influence of information communication technology (ICT)-integrated curriculum content, material, and teaching strategies on ICT competencies of students in blended learning. The ICT-integrated teaching strategies were used as a mediator in between the relationships of curriculum content, material, and ICT competencies. We used a survey questionnaire containing 26 items on the variables of research in this study. The data were collected from six universities in the Hunan Province of China. The target population consisted of undergraduate students of blended learning. In total, 486 participants participated in the study. Partial least squares-structural equation modeling (PLS-SEM) was applied to measure the relationships among variables. The results revealed that there were significant and positive relationships among ICT-integrated curriculum content, material, teaching strategies, and ICT competencies of students. Furthermore, it was also revealed that ICT-integrated teaching strategies in blended learning mediated the relationships in between ICT integrated curriculum content, material, and ICT competencies. It was concluded that the effective curriculum content, curriculum material, and teaching strategies are the critical predictors of ICT competencies. Moreover, teaching strategies worked as an intervening factor between the curriculum content, curriculum material, and ICT competencies. The practical implications and directions for future research are also presented in this study.

## Introduction

Blended learning has been around for a while and is defined as the combination of normal and online learning. It provides opportunities for students and teachers to share and discuss academic concepts and ideas through direct instruction and group discussion by using technology (Law et al., 2019). By nature, blended learning not only meets the learning needs of students but also focuses on the various learning preferences of students (Béres et al., 2012). There are some major components of blended learning such as the curriculum content, curriculum material (CM), teaching strategies (TS), and information communication technology (ICT), which play a pivotal role in its quality and success.

As all over the world, China has successfully implemented the concept of blended learning in its academia. Especially throughout the last decade, a significant progress has been observed in the field of blended learning in China (Pang et al., 2010; Lu et al., 2012; Zhang and Han, 2012; Anthony et al., 2019; Law et al., 2019; Gao et al., 2020). This study has been designed to examine the role of different components of blended learning such as curriculum content, CM, TS, and ICT competencies. The role is examined by determining the relationships among these components of blended learning in the Chinese context.

The curriculum content in blended learning is a component that provides sufficient subject matter for the learning of the students. The content of the curriculum in blended learning meets the needs and interests of the students (Savara and Parahoo, 2018). The curriculum content in blended learning should be flexible and easy to deliver by the teachers (Anthony et al., 2019). The students in a blended learning environment feel comfortable in their learning with the quality of curriculum, content design, and its delivery (Ozkan and Koseler, 2009). Furthermore, the curriculum is delivered using ICT resources such as We Chat Work, Zoom, Google Meet, and WhatsApp. Such a curriculum is called ICT-integrated curriculum, and it plays a significant role in the successful development of ITC competencies among students. Therefore, this study focuses on investigating the ICT-integrated curriculum content relationship with ITC competencies in blended learning.

Curriculum material comprised things that assist them in the delivery of curriculum and student learning as well. These material may include text books, lesson plans, study guides, handouts, or lecture notes (Kristanto, 2017). In blended learning, the CM is presented with the help of ICT such as PowerPoint presentation, voice notes of teachers, lecture recordings, and emails; such materials are called ICT-integrated CMs. Student shares their understanding regarding their work with their peers through online resources as face-to-face meeting facilitates the learning outcomes. CM solves several challenges through pedagogical and technological support in blended learning (Yao, 2019). Moreover, the teacher creates online group discussions and online assignment submissions through the material in hybrid courses (Lu et al., 2012). The ICT-integrated curriculum has made significant changes in the blended learning system. Moreover, CM should change focus from covering topics to integrated learning, which might improve IT competencies (Sabin et al., 2018). This analysis provides the insight related to the CM; thus, this study emphasizes exploring this construct in blended learning.

Similarly, TS are key components to establish and prepare blended learning through web-based instructional practices executed by teachers consistent with the dissimilar nature of different courses (Lu et al., 2012). TS require ICT knowledge and skills for better-blended learning to improve students learning outcomes. Such TS in which ICT components may include multimedia, laptop, notebook, CDs, still cameras, and e-books are called ICT-integrated teaching. Previous studies were given less focus in the area of TS with ICT competencies in blended learning in China (Anthony et al., 2019; Law et al., 2019; Gao et al., 2020). This research made an effort to explore the role of TS fostering the ICT competencies among the student.

Information communication technology plays an important and significant role in the successful launching of blended learning in any location. Without ICT, the program of blended learning is not possible. Due to this reason, it is very important for teachers and students to improve their ICT competencies for successful curriculum delivery and learning. ICT competencies, including web technology, software, hardware, and tools, are important outcomes of education in the technology era (Chang et al., 2006). An ICT competency-based curriculum needs to focus on developing professional ICT competencies that would support students at their workplace after their graduation. ICT competencies have been deemed one of the most important competencies across the professions (Cheng and Chau, 2016). We found fewer studies focused on assessing the ICT competencies development through blended learning courses among undergraduate students in China. This study tries to explore the ICT competencies of students enrolled in blended learning courses.

Despite the certain concerns among scholars and practitioners on effective curriculum content, CM, and TS in blended learning courses, various gaps have been observed in determining the interrelationship of curriculum content, TS, CM, and ICT competencies. Literature on blended learning was mainly produced in advanced countries, with research undertaken in the USA, Europe, Australia, and Japan. Meaningful research insights and significant findings are in their beginning stage for emerging nations like China. Most of the studies in blended learning have focused on students' behavioral aspects (Zhang J. H. et al., 2020). Less focus has been given to the curriculum content, TS, CM, and ICT competencies. Moreover, as per the authors' knowledge, a few studies have examined the direct and indirect relationship of curriculum content, CM, and TS with ICT competencies. Particularly, studies have not investigated TS as a mediator construct. The focus of this study was on exploring direct relationships of curriculum content and CM with ICT competencies. Furthermore, this study also explores indirect influence of the curriculum content and CM on ICT competencies through TS. The research framework was developed to addresses the following research questions:

**Research Questions 1:** How do the ICT-integrated curriculum content, teaching strategies, and CM influence the ICT competencies?
**Research Questions 2:** How do ICT-integrated teaching strategies play a mediating role in the relationship between the ICT-integrated curriculum content and ICT competencies?
**Research Questions 3:** How do ICT-integrated teaching strategies play a mediating role in the relationship between the ICT-integrated curriculum material and ICT competencies?

## Literature Review

### Blended Learning Research in China

Universities in China have raised their level of awareness in blended learning and have integrated the mode of blended learning in their educational setup (Zhang Y. et al., 2020). Furthermore, in the field of research in education, the theme of blended learning has emerged as a leading construct, especially in the current pandemic situation due to coronavirus disease 2019 (COVID-19) (Huang et al., 2020). Many researchers have discussed the theoretical and practical issues emerging during blended learning curriculum implementation and instructional strategies in China. Huang et al. (2008) discussed the design and theory of blended learning curriculum instruction and presented insights into the Chinese universities' context. The application of the curriculum content, material, and TS in blended learning for fostering ICT competencies are highly acceptable and acknowledged. However, the dominant roots of traditional teaching methods work in contradiction to the learning settings needed for typical blended learning in Chinese higher education (Tham and Tham, 2011). Chinese universities have started to offer courses in various fields through the blended learning mode (Gao et al., 2020; Yi, 2020). Moreover, qualified foreign faculty has developed various aspects to implement blended learning in the Chinese higher education system, especially in the last decade. This study was designed to examine the interrelationship among the curriculum content, material, TS, and ICT competencies in blended learning in China.

### ICT-Integrated Curriculum Content

The understanding about the curriculum content in blended learning has been developed in previous studies such as curriculum content in the teaching-learning process considered important facts, principles, and concepts that can be knowledge, skills, attitude, and values that students expose to learn (Lunenburg, 2011a,b). Koch et al. (2020) identified the significance of the blended learning curriculum of bioscience and provided students' insights into what they reflected as good and inadequate approaches to learning. Yu and Du (2019) explained the delivery of the curriculum content through tremendous advancements in technology that have become central points in modern higher education for both face-to-face learning and online learning. The use of ICT in the delivery of curriculum content has made significant advancements in blended learning. Therefore, curriculum contents in blended learning should pay more attention on integrative learning environments for better learning outcomes, which may develop ICT competencies among the students (Sabin et al., 2018). This study also focuses on the development of ICT competencies through ICT-integrated curriculum content in blended learning.

### ICT-Integrated Curriculum Material

Curriculum material in blended learning comprised textbooks, lesson plans, lecture notes, ppt presentations, learning managed system (LMS), software, web-based learning, and some other instructional material that supports the students in solving problems in blended learning courses (Sledgianowski et al., 2017; Yao, 2019). Numerous studies have investigated the effective use of digital CM, technologies, and resources for various academic disciplines (Drijvers et al., 2016; Pepin et al., 2017). It has also been discussed that CM and teaching methods have been broadened alongside the rapid development of IT technology and Internet networking in blended learning courses (Cheng and Chau, 2016). However, fewer researchers investigate the CM role in blended learning. This study examines the role of the ICT-integrated CM in improving ICT competencies.

### ICT-Integrated Teaching Strategies

Teaching strategies are a generalized lesson plane that includes the structure of desired outcomes in terms of goals, the outline of planned activities, and strategies for achieving desired goals (Suman and Tiwari, 2021). TS work as a central pillar of blended learning courses due to the hybrid nature in higher education (Smith and Hill, 2019). The TS for blended learning create a more flexible learning environment for student engagement than fully online or entirely on-site instruction (Hrastinski, 2019). TS reduce adverse outcomes on student engagement, motivation, and academic success through the flexible nature of instruction (Gleason and Greenhow, 2017; Ibrahim and Nat, 2019). ICT has made teaching very effective in blended learning, and the teachers use various tools of ICT to make their teaching effective such as multimedia, digital communication platforms like Google Meet, We Chat, Zoom, and WhatsApp. Educational institutions are investing in technology-enhanced learning, which raises the question of how these approaches shape the teaching process effectiveness in hybrid or blended learning environments (Hrastinski, 2019). TS through blended learning are fast growing as reasonable and substitute approaches to increase course registrations and improve educational outcomes in universities (Gleason and Greenhow, 2017). Thus, this study examines the role of ICT-integrated TS in improving ICT competencies among students enrolled in blended learning courses.

### ICT Competencies

Information communication technology competencies represent the skills and knowledge that support the practical operations expected from the teachers and students in an educational organization (Jabbarova, 2020). ICT competencies have a unique role in performing the organizational process at the workplace (Murawski and Bick, 2017). Higher education institutions are concentrating on offering various computing programs at different levels to improve the ICT competencies of professionals (Husam et al., 2018). Chae et al. (2018) explained the ICT competencies role in strategic decision-making among ICT leaders in the organization and highlighted the significance of ICT competencies for professionals. The curriculum content, TS, and CM are also the main ingredients in higher education to enhance learning ICT competencies (Sabin et al., 2018). Therefore, the effectiveness of these ingredients is supposed to be assessed for local understanding, especially among the students enrolled in blended learning courses. This study is an effort to investigate the role of the curriculum content, material, and TS in fostering the ICT competencies of students in blended learning.

Blended learning has emerged as a growing trend during the pandemics. Researchers have studied different behavioral aspects of blended learning such as attitude and intentions (Asghar et al., 2021a,b,d), but there is a scarcity of studies related to ICT competencies in blended learning approaches (Asghar et al., 2021c, 2022). It needs to study the influence of ICT-integrated content, strategies, and teaching material for ICT competencies in blended learning approaches beyond the discussion of behavioral aspects (Ashraf et al., 2021). Because these are essential components to develop ICT competencies (Sabin et al., 2018), less studies have found the direct relationship of the content and teaching materials with ICT competencies development (Urquijo and Natalio, 2017). Especially, TS play an essential role for teaching content delivery for ICT competencies development. Moreover, there is a gap in the research to find the mediation of TS between the ICT content, material, and ICT competencies development.

## Research Framework

**Figure 1:**
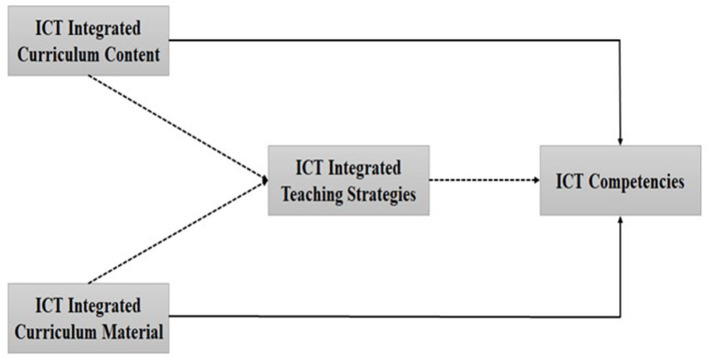
Research framework. Dotted lines show hypothesized indirect relationships. Solid lines show the direct relationship between constructs used in the framework.

The extensive review of related literature enabled the researchers to develop an operational research framework for the purpose of the study. The research framework highlighted the interrelationships among the variables such as the curriculum content, material, TS, and ICT competencies. It has been mentioned earlier that the curriculum content, material, and TS in blended learning are considered to be predictors of ICT competencies among students (Sabin et al., 2018). The review of the literature suggests that the curriculum content, material and TS influence the ICT competencies (Papanikolaou et al., 2017; Chae et al., 2018; Husam et al., 2018; Apandi and Raman, 2020). Therefore, this study intends to examine the interrelationships among the variables of the study such as the curriculum content, material, TS, and ICT competencies. Furthermore, direct relationships among these variables were sorted out in this study. The intervening or mediating role of TS in strengthening these relationships was the major area of interest in this study. This research framework was implemented and evaluated for blended learning set up in the Chinese context (See Figure 1).

## Hypotheses Formulation

### ICT-Integrated Curriculum Content and ICT Competencies

An effective curriculum content is considered the potential factor to improve information technology competencies (Vreeburg Izzo et al., 2010). In contrast, it was found that the curriculum content requires potential changes for improvements of ICT competencies (Mohan et al., 2014). Kircaburun and Griffiths (2018) measured the curriculum content effects on ICT outcomes and found that the curriculum content improved ICT competencies. Lei et al. (2018) investigated the opportunities and challenges of the ICT curriculum in library and information science programs and found that content for improving ICT competencies was missing and recommended for the improving ICT competency-based curriculum. Northey et al. (2018) measured the current state of information technology curriculum effectiveness for ICT competencies and found that the ICT curriculum content has a limited capacity to produce required skills among students. Lei et al. (2018) conducted studies in the Australian context and found that the ICT curriculum is inadequate for improving graduates' ICT competencies for their employment. It was assumed that the effective curriculum content is a predictor of IT competencies through blended learning courses. We could find no study measuring the relationship between curriculum content and ICT competencies in the Chinese context. Therefore, the positive relationship between curriculum content and ICT competencies was predicted in the following hypothesis:

**Hypothesis 1:** ICT-integrated curriculum content has a positive relationship with ICT competencies.

### ICT-Integrated Curriculum Material and ICT Competencies

With the extensive literature review, we found the gap in the literature, that is, CM's role in improving ICT competencies (Boyatzis et al., 2017). (Zhoc et al., 2020) conducted an ICT competency-based assessment and presented a curriculum model to guide the curriculum designers in improving information technologies competencies. Thomas et al. (2017) explored CM links with the website-based teaching in information technology education and suggested some reforms in CM for ICT education in China. This area of the research provoked the attention of the researchers to include this component of the curriculum in the research framework in the study for further investigating the nexus of CM and ICT competencies in blended learning in China. Therefore, the study examined the relationship between ICT integrated CM and ICT competencies in the following hypothesis:

**Hypothesis 2:** ICT-integrated curriculum material has a positive relationship with ICT competencies.

### ICT-Integrated Teaching Strategies and ICT Competencies

Teaching strategies play a very significant role in improving ICT competencies (Lei et al., 2018). MacCann et al. (2020) applied technological pedagogical content knowledge theory to assess practical teaching to enhance ICT competencies and found that this framework can be helpful to develop ICT skills among students. Yusuf (2005) explored TS with the information technology content and tool to improve ICT skills among students and concludes that TS with information technology content and tools increase ICT skills among nursing students. Similarly, another study by Teo (2008) investigated the role of the ICT knowledge patterns among medical students in a university teaching hospital in Nigeria, and results indicate the need for more structured teaching and training to build the ICT knowledge pattern. Urquijo and Natalio (2017) explored the TS for ICT competencies development among music students. The results indicated that ICT pedagogy was effective for modern musical computer skills. However, a lot of studies are available which examined the relationship between TS and ICT competencies. However, minimum research has been conducted to examine the influence of ICT integrated teaching on the development of ICT competencies among students; especially, no research has been presented to determine the impact of ICT integrated teaching on the development of ICT competencies among students in blended learning set up of China. Based on this rationale in the field of education and research, we assumed that ICT-integrated TS have a positive relationship with ICT competencies among students enrolled in blended learning courses in the following hypothesis:

**Hypothesis 3:** ICT-integrated teaching strategies have a positive influence on ICT competencies.

### The Mediating Role of ICT Integrated Teaching Strategies Between Curriculum Content and ICT Competencies

Most studies indicate that the curriculum content plays a significant role in developing the ICT competencies of students (Vreeburg Izzo et al., 2010). Some of the studies assessed the strengths and weaknesses of the ICT integrated curriculum and explored that curriculum content supports ICT competencies (Lei et al., 2018). On the other hand, some studies indicated that the curriculum content has a role to improve ICT competencies and required changes and guide the curriculum designers to develop ICT competencies-based CM in the Chinese context (Zhoc et al., 2020). The role of TS as a mediator construct between the curriculum content and ICT competencies is yet to explore among the students enrolled in blended learning courses in China. Therefore, this study investigates the mediating role of TS in strengthening the relationship between the curriculum content and ICT competencies. The research model proposed for this study shows the tentative or expected mediation of ICT-integrated TS in between the relationship between ICT-integrated curriculum content and ICT competencies. The following hypothesis was formulated based on this discussion:

**Hypothesis 4:** ICT-integrated TS have a positive mediating role between the relationship of curriculum content and ICT competencies.

### The Mediating Role of ICT-Integrated Teaching Strategies Between Curriculum Material and ICT Competencies

Various studies suggested that the nexus of CM and IT competencies are needed to be explored (Boyatzis et al., 2017). Moreover, the role of TS in improving IT competencies is much important in various fields (Lei et al., 2018). Urquijo and Natalio (2017) investigated the positive role of TS to improve the IT competencies among music students. The role of TS as a mediator construct between CM and ICT competencies is yet to explore among the students enrolled in blended learning courses in China. Therefore, this study investigates the mediating role of TS in strengthening the relationship between the CM and ICT competencies. The research model proposed for this study shows the tentative or expected mediation of ICT-integrated TS in between the relationship ICT-integrated CM and ICT competencies. The following hypothesis was formulated based on this discussion:

**Hypothesis 5:** ICT-integrated teaching strategies have a positive mediating role between the relationship of CM and ICT competencies.

## Research Methods

This study is a part of a large project supported by the National Natural Science Foundation of China (Grant No. 71950410624), focused on investigating the role of the internet and technology in blended learning in Chinese academia. Currently, China has experienced much progress in blended learning. Previously, most studies focused on blended learning have been conducted in advanced countries, while very limited studies have been conducted with different blended learning contexts in Chinese education. Due to this emerging trend of blended learning in China, the researchers inclined their attention toward examining the interrelationship among ICT-integrated curriculum content, material, ICT-integrated TS, and ICT competencies. The study was descriptive in nature; furthermore, it was a relationship study. The study was accomplished by adopting the following methodology and procedure.

### Questionnaire Design

**Figure 2:**
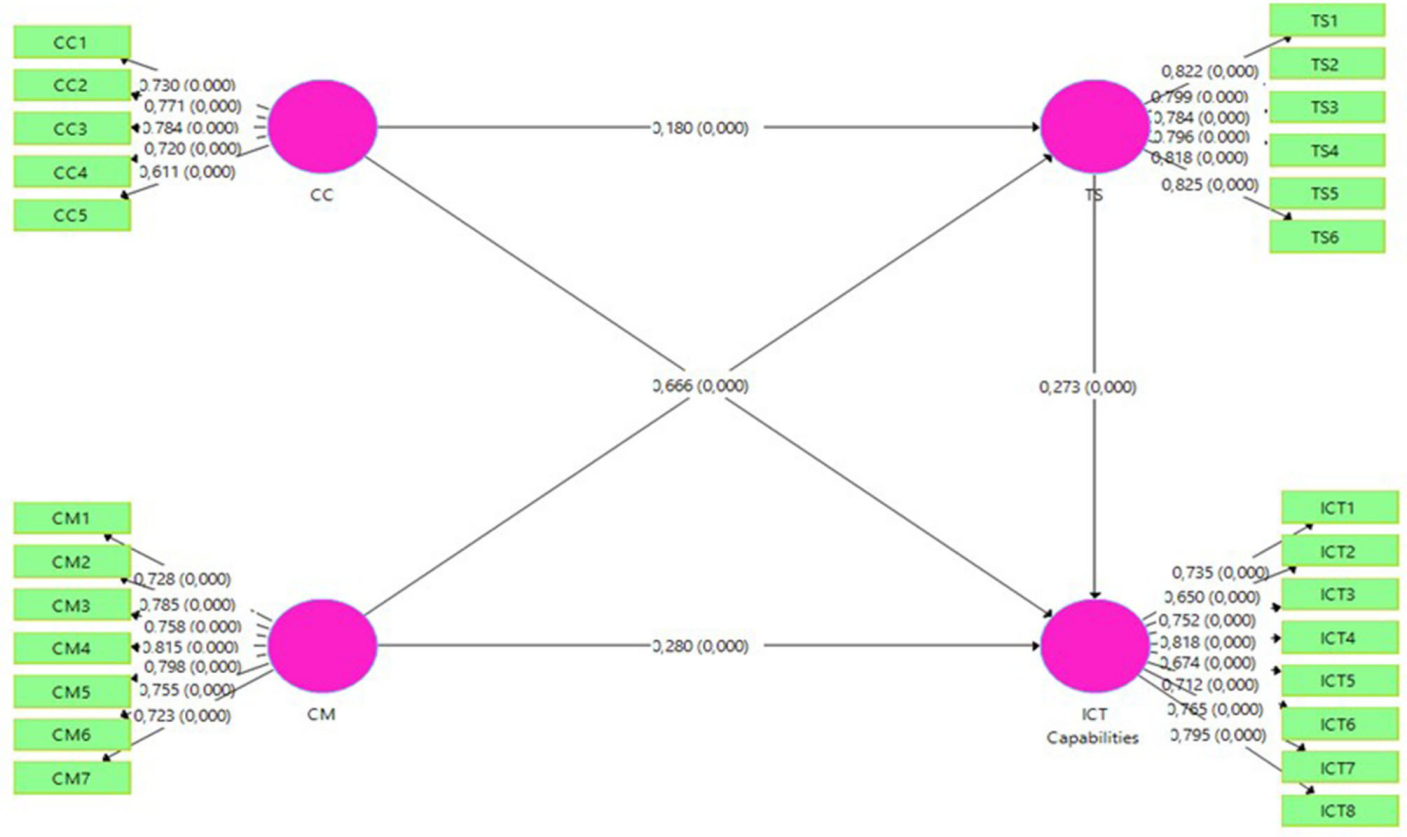
The theoretical constructs with *R*^2^ values.

A survey questionnaire was used to collect data in this study. The questionnaire comprised 26 items. The responses were captured on a 7-point Likert scale. The scale items were developed by extracting the themes from the literature review. There were five, six, seven, and eight items, respectively, for the variables of the study such as the curriculum content, material TS, and ICT competencies. We conducted a pilot study on 20 participants having similar characteristics as the final participants to check the validity and reliability of the questionnaire. Minor modifications were made based on the participant feedback. It was ensured that all items used in the questionnaire were well-understood and the participants filled out successfully. Moreover, the detail of the questionnaire and indicator loading of the items are presented in **Table 2** and Figure 2.

### Measures

#### ICT-Integrated Curriculum Content

The items related to the curriculum content (CC) were adapted from the work of TLEMU-HKU (2018). CC comprised seven items marked on the 7-point Likert scale ranging from 1 strongly disagree to 7 strongly agree. Examples of items included “I have acquired sufficient content knowledge about ICT competencies” and “I know concepts, facts, theories, and procedures within the information technology contents.” The Cronbach's alpha (CA) for curriculum content was 0.772 (**Table 2**).

#### ICT-Integrated Curriculum Material

The items related to the CM were adapted from the work of TLEMU-HKU (2018). CM comprised seven items marked on the 7-point Likert scale, 1 strongly disagree to 7 strongly agree. Examples of items included “The material I used in the class was meaningful for me in using information technology” and “The material about information technology I used in the class induced interest and motivation in me.” The CA for the CM was 0.883 (**Table 2**).

#### ICT-Integrated Teaching Strategies

The items related to the TS were adapted from the work of TLEMU-HKU (2018). TS comprised seven items marked on the 7-point Likert scale, ranging from 1 strongly disagree to 7 strongly agree. Example items were included as “meaningful interaction between students and teachers beyond the classroom are ensured” and “our teacher provides me a conducive information technology learning environment.” The CA for TS was 0.893 (**Table 2**).

#### ICT Competencies

We adapted items related to the ICT competencies were adapted from the work of TLEMU-HKU (2018). ITC comprised seven items marked on the 7-point Likert scale, 1 strongly disagree to 7 strongly agree. Examples of items included “I have basic technology managing competencies” and “I know the use of information systems which help integrate the institutional operations.” The CA for ICT competencies was 0.881 (**Table 2**).

### Data Collocation

The data were collected from undergraduate students enrolled in business and management sciences programs in six universities located in the Hunan Province from September 2019 to December 2019. However, the data analysis and data presentation were delayed due to the COVID-19 pandemic. Students who were enrolled in the courses that used the blended learning approach were invited only to fill the questionnaire. We used pseudonyms for selected universities to keep confidentiality. Before data collection, ethical approval was obtained from the ethics committee of Hunan University, and the research protocol was carried out by following the rules of the Declaration of Helsinki of 1975 revised in 2013. All participants gave their consent before participating in the research. We distributed 500 questionnaires among students, 492 were returned, while six questionnaires were rejected due to incomplete information. The final sample consisted of 486, with a return rate of 97.2%.

### Demographics

The selected universities were already offering blended learning courses and were aware of the importance of IT competencies through curriculum content, CM, and TS. The majority of the respondents were female (male 25.7% and female 74.3%); 57.4% respondents were from urban and 42.6% were from a rural area; having age 18–20 years were 33.5%, 21–23 years were 56.2%, and above 24 years were 10.5%; from 1st year were 32.1%, 2nd year were 37.0%, 3rd year were 16.3%, and 4th year were 14.6%. The sample size was considered appropriate to run a path analysis in SmartPLS. Prior research studies have had relatively similar sample characteristics (Iqbal et al., 2021). The respondents' sample demographic statistics are presented in Table 1.

**Table 1:** Demographic statistics.

**Measures**	**Items**	**Frequency (*n*)**	**Percentage %**
Gender	Male	125	25.7
	Female	361	74.3
	Total	486	100
Location	Rural	207	42.6
	Urban	279	57.4
	Total	486	100
Age	18–20	163	33.5
	21–23	273	56.2
	24 to onwards	50	10.5
	Total	486	100
Years of Education	1st year	156	32.1
	2nd year	180	37.0
	3rd year	79	16.3
	4th year	71	14.6
	Total	486	100

## Results

### Confirmatory Factor Analysis

In this study, we applied confirmatory factor analysis (CFA) to measure the reliability and validity of the instrument (Tasdemir et al., 2020a). This data analysis method deals with the structural equation modeling (SEM). The convergent and discriminant validity of all constructs were determined to check the measurement model's overall fitness (Zhou et al., 2021). The model fitness was active to enhance up to the proposed level. Few items were removed after performing several trials to reach the suggested scale levels. The standard value for indicator loading (IL), CA, rho-A, and composite reliability (CR) is more than 0.70, while average variance extracted (AVE) is more than 0.50 (Hair et al., 2019). Table 2 shows that all indicators (i.e., IL, CA, and CR) are more than 0.70 (Iqbal et al., 2022a), while AVE is >0.50. The factor loading standard value of more than 0.50 is acceptable if AVE is >0.50 (Iqbal et al., 2021).

**Table 2:** Reliability and convergent validity.

**Sub-Scales**	**IL**	**CA**	**rho_A**	**CR**	**AVE**
Curriculum content		0.772	0.776	0.847	0.527
CC1	0.712				
CC2	0.770				
CC3	0.784				
CC4	0.720				
CC5	0.611				
Curriculum material		0.883	0.885	0.909	0.588
CM1	0.728				
CM2	0.785				
CM3	0.758				
CM4	0.815				
CM5	0.798				
CM6	0.755				
CM7	0.723				
Teaching strategies		0.893	0.894	0.918	0.652
TS1	0.822				
TS2	0.799				
TS3	0.784				
TS4	0.796				
TS5	0.818				
TS6	0.825				
ICT competencies		0.881	0.887	0.906	0.547
ITC1	0.735				
ITC2	0.650				
ITC3	0.752				
ITC4	0.818				
ITC5	0.674				
ITC6	0.712				
ITC7	0.765				
ITC8	0.795				

*IL, indicator loading; CA, Cronbach's alpha; CR, composite reliability; AVE, average variance extracted; IT, information technology*.

Recently, Henseler et al. (2015) analyzed Zhoc et al.'s (2020) measurement approach and suggested this approach is not appropriate for measuring discriminant validity. Henseler et al. (2015) presented another technique of Heterotrait-Monotrait (HTMT) to assess discriminant validity. HTMT is described as the item correlations mean values across the constructs relative to the (geometric) mean of the average correlations for the items measuring the same construct (Hair et al., 2019). In this study, we applied the HTMT technique to measure the discriminant validity. Henseler et al. (2015) suggested the standard value for HTMT as 0.90. The HTMT value is higher than 0.90, which means the discriminant validity is not present. Table 3 shows that the HTMT value of each construct is <0.90, which means the scale justified the discriminant validity requirements.

**Table 3:** Convergent and discriminant validity.

**Sub Scales**	**AVE**	**Curriculum content**	**Curriculum material**	**IT competencies**	**Teaching strategies**
Curriculum content	0.527	0.725			
Curriculum material	0.588	0.556	0.767		
ICT competencies	0.547	0.474	0.583	0.740	
Teaching strategies	0.652	0.546	0.765	0.580	0.807

*HTMT, Heterotrait-Monotrait; AVE, average variance extracted*.

This study tests the collinearity problem through SEM and ensured that collinearity problems have resolved. The threshold value for variance inflation factor (VIF) is <5 (Huang, 2021). The VIF value in SEM is <5 and is ranging between 1.436 and 2.568, which indicates that there is no collinearity problem among the study dimensions. This study tests the model fit through three main indicators, namely, SRMR, NFI, and RMS_theta for PLS-SEM. The threshold value for SRMR is between 0 and 1 (Tasdemir et al., 2020b), and <0.08 is considered an ideal value for the good fit of the model (Bentler and Bonett, 1980). The threshold value for NFI is between 0 and 1. The ideal value for NFI is above 0.90 (Bentler and Bonett, 1980). The threshold value for RMS_theta is <0.12. The RMS_theta value is the most suitable for assessing the reflective measurement models (Henseler et al., 2014). The SRMR value for model assessment is 0.061. NFI has a value of 0.863. The RMS_theta has 127. The NFI value of 0.863 is <0.90 but is not much different and acceptable (Huang, 2021). Therefore, it is concluded that the model was reasonably well-fitted in general in this research. The analysis of collinearity and model fit is presented in Table 4.

**Table 4:** Collinearity analysis and model fit.

**Dimensions**	**VIF-ICT capabilities**	**VIF-TS**	**Model fit**	
CC	1.520	1.437	SRMR	0.061
CM	2.568	1.436	NFI	0.863
TS	2.552		rms Theta	0.127

We evaluated the explanatory power of the model on the basis of the *R*^2^ value. The *R*^2^ value range is between 0 and 1. The threshold value of *R*^2^ for explanatory power is near 0.75, 0.50, and 0.25, which has a strong, moderated, and weak explanatory power, respectively (Iqbal et al., 2022b). Table 5 can be seen that TS have a strong explanatory power, while the ICT competencies have moderated explanatory power. Therefore, the model explains the latent variables very well, and it has a good degree of explanatory power.

**Table 5:** R-square value.

**Latent variables**	**R square**	**R square adjusted**
ICT Capabilities	0.402	0.398
TS	0.608	0.606

### Descriptive Statistics

The survey respondents were analyzed using descriptive statistics displayed in Table 6. As mentioned earlier, the responses were taken on a 7-point Likert scale. The range of mean scores of responses is 4.1468–5.0851. The standard deviation range was recorded from 0.99349 to 1.38127.

**Table 6:** Descriptive statistics.

**Constructs**	***N***	**Minimum**	**Maximum**	**Mean**	**Std. deviation**
ICT Competencies	486	1.00	7.00	4.1468	0.99349
Curriculum Content	486	1.00	7.00	4.8246	1.11995
Curriculum Material	485	1.00	7.00	5.0851	1.36053
Teaching Strategies	486	1.00	7.00	5.0507	1.38127

### Regression Analysis

We applied regression analysis through SmartPLS 3.2.2 to explore the associations drawn in the conceptual framework (Hair et al., 2019). The variance-based structural equation modeling (VB-SEM) method was applied, which enabled concurrent evaluation of the measurement model (Malik et al., 2021). This approach analyzes the reliability and validity of the items used in conceptual variables with the structural modeling of the hypothesized relationship among constructs involved in the model (Zhoc et al., 2018; Hair et al., 2019). Table 5 exhibits the direct effect of ICT-integrated curriculum content on ICT competencies. Results revealed that ICT-integrated curriculum content has a significant and positive relationship with ICT competencies (β = 0.169, *p* < 0.05), which supports hypothesis H1. Furthermore, ICT-integrated CM has a significant and positive relationship with IT competencies (β = 0.280, *p* < 0.05), which approved hypothesis H2. Similarly, ICT-integrated TS have a significant and positive relationship with ICT competencies (β=0.273*, p* < 0.05), which approved hypothesis H3. Moreover, in an indirect relationship, TS mediate between the curriculum content and ICT competencies. Results indicate that ICT-integrated TS mediated the positive and significant relationship between ICT-integrated curriculum content and IT competencies (β = 0.049, *p* < 0.05), which supports hypothesis H4. Finally, ICT-integrated TS mediated the significant and positive relationship between ICT-integrated CM and ICT competencies (β = 0.182, *p* < 0.05), which approved hypothesis H5 (Table 7).

**Table 7:** Direct and indirect relations.

**Direct relations**	**Estimations**	**Mean**	**Standard deviation**	**T statistics**	***P*** **-values**
CC -> ICT capabilities	0.169	0.172	0.047	3.592	0.000
CM -> ICT capabilities	0.280	0.278	0.063	4.474	0.000
TS -> ICT capabilities	0.273	0.274	0.06	4.542	0.000
**Indirect relations**					
CC -> TS -> ICT capabilities	0.049	0.050	0.015	3.348	0.001
CM -> TS -> ICT capabilities	0.182	0.182	0.042	4.366	0.000

*CC, curriculum content; IT, information technology; CM, curriculum material; TS, teaching strategies*.

## Discussion

This study determines the meaningful relations based on the synthesized theoretical framework. Prior work in a similar area has been focused on advanced countries (Nelis et al., 2011; Kaur et al., 2018; Li and Xu, 2019), while very few studies have been conducted in emerging nations like China (Maguire et al., 2017; Li and Xu, 2019). Moreover, these limited studies have revealed a lack of attention to the curriculum contents in blended learning while paying more attention to the substantial roles for efficient use of resources in blended education. To the best of researchers' understanding, this research is the first to explore the impact of curriculum content and material on ICT competencies in the context of blended learning courses in higher education, especially considering TS as a mediating construct.

This study measures the direct relationship of curriculum content with ICT competencies. Results showed that the curriculum content positively influences the ICT competencies, which approved our hypothesis H1. Previous studies discussed the curriculum content relationship with ICT competencies (Howard et al., 2018). Dede (2008) explored the relationship between the curriculum content and ICT competencies. The results revealed that there is a strong positive association between the ICT-integrated curriculum content and ICT competencies of students in blended learning. It might be happened due to the integration of ICT in the curriculum content and subject matter of the courses studied in blended learning. The learning through ICT in the curriculum content enables the students to furnish their ICT competencies.

Furthermore, this research concentrated on the direct association of CM on ICT competencies. Results indicated that the CM positively influences the ICT competencies, which supports our hypothesis H2. Prior studies show that CM has a positive relationship with ICT competencies (Sledgianowski et al., 2017). (Mohan et al., 2014) conducted a survey in health sector professionals and found that the CM needs revisions to meet the professionals' expectations. Our study produced different results. The data analysis revealed that there is a strong positive association between ICT integrated curriculum content and ICT competencies of students in blended learning. It might happened due to integration of ICT in the CM of the courses studied in blended learning. The learning through ICT in the CM enables the students to improve their ICT competencies.

In addition, this study measures the direct relationship of TS with ICT competencies. Results showed that TS positively influence the ICT competencies, which approves our hypothesis H3. Previous studies show that TS positively affect ICT competencies (Shieh and Yu, 2016; Obermayer et al., 2017). Dede (2008) explored the relationship between TS and ICT competencies. The data analysis revealed that there is a strong positive association between ICT TS and ICT competencies of students in blended learning. It might be happened due to integration of ICT TS to teach courses in blended learning. The learning through ICT-integrated teaching enables the students to develop their ICT competencies.

The study also investigated the indirect relationship of TS between the curriculum content and ICT competencies. Results reflected that the TS mediated between curriculum content and ICT competencies, which supports hypothesis H4. Past literature on this area confirms that TS mediated between curriculum content and ICT competencies (Lawson et al., 2014). Sledgianowski et al. (2017) described how instructional resources facilitate ICT competencies and explained that TS are a significant source of developing ICT competencies in students. In this study, we concluded that TS play a positive and intervening role in facilitating the curriculum content delivery for ICT competencies development among students.

Finally, this study examines the indirect relationship of ICT-integrated TS among ICT-integrated CM and ICT competencies. Results reflected that the TS mediated between ICT-integrated CM and ICT competencies, which approves hypothesis H5. Past literature on this area mentioned that TS strengthen the association between CM and ICT competencies (Chien et al., 2014; Ghavifekr and Rosdy, 2015). Mohan et al. (2014) explained that up-to-date TS with CM related to ICT competencies. Therefore, it was concluded that TS effectively work with the CM to enhance ICT competencies among students enrolled in blended learning courses.

## Conclusion

The conceptual model of this research was developed based on the insights offered by the existing literature on technology integration in blended learning. Our results confirm the relationship between the curriculum content, material, and TS and ICT competencies among students enrolled in blended learning courses. The findings of this study support that TS have a direct positive and significant effect on ICT competencies. Curriculum content and material have a direct positive and significant influence on ICT competencies. Moreover, results confirm that TS have an intervening role between the curriculum content, material, and ICT competencies.

Our results can also be interpreted as follows: ICT-integrated curriculum content, material, and TS have a key role in developing ICT competencies. Curriculum content and material are significant factors for developing ICT competencies among students enrolled in blended learning courses. Similarly, effective TS are the key predictors of ICT competencies. However, TS with ICT CM have an interventional role with curriculum content and material to enhance ICT competencies. It was not beyond our conclusions that the results of the study encompass the major assumptions of the researchers, which were about the interrelationships among ICT-integrated curriculum content, material, and TS and ICT competencies, which indicates that all three independent variables of the study such as ICT integrated curriculum content, material, and TS worked as predictor variables in the study. The dependent variable of the study, which was the ICT competencies of the students, proved to be criterion variables in the study. It is worthwhile to mention in this study that the presumed intervening relationship of TS in strengthening the relationship among ICT-integrated curriculum content, material, and ICT competencies proved to be true. Finally, it can be deduced from the results of the study that ICT-integrated TS significantly and positively mediate the association of ICT-integrated curriculum content and material with ICT competencies of the students in the blended learning set up in China.

## Implications

Effective curriculum content delivery, TS, and CM are significant predictors in fostering the ICT competencies of students in blended learning. Universities are trying to promote blended education by introducing blended learning courses, which improve ICT competencies among students at various levels. Universities are required to frequently update their curriculum content and material to improve ICT competencies. The curriculum designer may take insights from the findings of this research to develop curriculum content for other programs. TS are considered important factors for improving ICT competencies. Therefore, teachers need to continue working to apply updated TS to improve ICT competencies effectively. TS can strengthen the relationships among curriculum content, material and ICT competencies. Therefore, teachers and curriculum designers can take guidelines from the finding of this study to create the alignment between these factors to improve ICT competencies. Furthermore, the implication of this study can be addressed in blended learning set in the Chinese context in the ways that the students should be provided with maximum opportunities to learn through ICT-integrated curriculum content, material, and TS for the development of their ICT competencies.

### Limitation and Future Research

Despite of its substantial implications, our study has some limitations that may affect the generalization of the findings. First, the participants came from six universities only located in one province, which might indicate a cultural bias to the scope of its findings. The validations of the results based on empirical data from other parts of China as well as from other emerging nations are needed. Second, we collected the data from the students who are enrolled in undergraduate programs only, which may bias generalizing results on students from other levels. Therefore, future research may include the participants who are from graduate or postgraduate. Third, we collected data undergraduate students enrolled in business and management sciences programs only. The social science, natural science, and medical science students were not part of this study sample. Therefore, it is recommended that future studies can include the participants from social science, natural science, and medical students, which might be helpful for validation of results from other disciplines. Future research would be interesting to explore cognitive and behavioral engagement as mediators between curriculum content with TS and ICT competencies.

## Data Availability Statement

The raw data supporting the conclusions of this article will be made available by the authors, without undue reservation.

## Ethics Statement

The studies involving human participants were reviewed and approved by Ethics Committee of Hunan University. The patients/participants provided their written informed consent to participate in this study.

## Author Contributions

MAsh and JI: conceptualization. JI: formal analysis, and software. JI, MAsh, MAr, and MAsg: methodology. JI, MAr, and MAsg: resources and writing original draft preparation. MAsh, MAr, and MAsg: writing review and editing. MAsh: funding acquisition. All authors have read and agreed to the published version of the manuscript. All authors contributed to the article and approved the submitted version.

## Funding

This research was supported by the National Natural Science Foundation of China (The Research Fund for International Young Scientists, Grant no. 71950410624).

## Author Disclaimer

Opinions reflect those of the authors and do not necessarily reflect those of the grant agencies.

## Conflict of Interest

The authors declare that the research was conducted in the absence of any commercial or financial relationships that could be construed as a potential conflict of interest.

## Publisher's Note

All claims expressed in this article are solely those of the authors and do not necessarily represent those of their affiliated organizations, or those of the publisher, the editors and the reviewers. Any product that may be evaluated in this article, or claim that may be made by its manufacturer, is not guaranteed or endorsed by the publisher.
